# Acute Consumption of Resistant Starch Reduces Food Intake but Has No Effect on Appetite Ratings in Healthy Subjects

**DOI:** 10.3390/nu9070696

**Published:** 2017-07-04

**Authors:** Jorge L. Ble-Castillo, Isela E. Juárez-Rojop, Carlos A. Tovilla-Zárate, Carlos García-Vázquez, Magda Z. Servin-Cruz, Arturo Rodríguez-Hernández, Claudia I. Araiza-Saldaña, Ana M. Nolasco-Coleman, Juan C. Díaz-Zagoya

**Affiliations:** 1 Centro de Investigación, DACS, Universidad Juárez Autónoma de Tabasco (UJAT), Villahermosa, 86150 Tabasco, Mexico; iselajua22@yahoo.com.mx (I.E.J.-R.); gbasecs@hotmail.com (C.G.-V.); zulemyservin_1990@hotmail.com (M.Z.S.-C.); ivonne-araiza@hotmail.com (C.I.A.-S.); zagoya@unam.mx (J.C.D.-Z.); 2 División Académica Multidisciplinaria de Comalcalco, UJAT, Comalcalco, 86658 Tabasco, Mexico; alfonsotovillaz@hotmail.com; 3 Unidad de Medicina Familiar 10, Instituto Mexicano del Seguro Social (IMSS), Xalapa, 91000 Veracruz, Mexico; rodheart@hotmail.com; 4 Unidad de Medicina Familiar 39, IMSS, Villahermosa, 86070 Tabasco, Mexico; anacoleman1@hotmail.com

**Keywords:** native banana starch, resistant starch, appetite hormones, visual analogue scale, glycemia, insulin

## Abstract

Previous studies have shown the benefits of native banana starch (NBS) supplementation in improving glucose metabolism and reducing body weight (BW) in humans. However, the effect of this starch on appetite regulation is unknown. The aim of this study was to examine the effects of NBS rich resistant starch on subjective measurements of appetite, energy intake, and appetite hormones in healthy subjects. Postprandial glucose and insulin responses were also assessed. In a randomized, single-blind, crossover study, 28 healthy young subjects consumed a beverage containing either 40 g of NBS or 40 g of digestible corn starch (DCS) on two separate occasions. Effects on appetite were estimated using visual analogue scales (VAS) and satiety hormone responses. At the end of the intervention, participants were provided with a pre-weighed ad libitum homogeneous test meal. After a washout period of 1 week, subjects received the alternative treatment. NBS supplementation induced a reduction in food intake, glucose area under the curve (AUC)-180 min, and insulin AUC-180 min. However, there was no associated effect on the subjective appetite ratings or gut hormones. NBS supplementation may help to reduce meal size and control BW.

## 1. Introduction

Obesity has become a global epidemic. The combined prevalence of overweight and obesity rose by 27.5% for adults and by 47.1% for children between 1980 and 2013. Mexico is among the countries with the highest prevalence of obesity in both adulthood and childhood [1]. The ongoing epidemic has led researchers to focus on finding novel ways to prevent weight gain or to reduce body weight (BW). This goal is especially important considering that obesity is one of the major risk factors for type 2 diabetes (T2D) and cardiovascular disease (CVD). 

Epidemiological studies show that fiber intake is inversely associated with BW [2,3,4]. Fiber is defined as a nondigestible carbohydrate and lignin that is intrinsic and intact in plants and also includes nondigestible carbohydrates that have beneficial physiological effects in humans [5]. Fiber consumption has been proposed to contribute to weight loss through several mechanisms, including the alteration of gut motility, the attenuation of nutrient absorption, and the lowering of overall caloric intake [2,6]. In addition, fiber consumption may also enhance satiety through other mechanisms, such as adding bulk and viscosity to the intestinal content and altering the secretion of gut hormones that influence metabolism and energy expenditure [7,8]. Improved satiety and reduced food intake are common theories used to describe why fiber intake may be associated with a lower body weight [9]. 

In particular, resistant starch (RS) is a nonviscous, fermentable fiber defined as the portion of starch that cannot be digested by amylases in the small intestine and that passes to the colon to be fermented by microbiota [10]. There are currently five types of resistant starch: RS1, starch granules surrounded by indigestible plant material such as protein matrices and cell walls; RS2, native granular starch, such as that found in green banana starch, raw potatoes, and high-amylose maize starch; RS3, crystallized starch made by alternative cooking-cooling; RS4, chemically modified starch formed either by cross-linking or by adding chemical derivatives; and RS5, starch interacting with lipids, amylose, and long-branch chains of amylopectin that forms single-helical complexes with fatty acids and fatty alcohols [11]. RS is known to exert important physiological effects that go beyond resistance to digestion, including modulation of satiety perception, dyslipidemia, insulin sensitivity, and glycemic control in healthy, obese subjects and in patients with metabolic syndrome [12].

The effects of RS on glycemic response have been explained by its low rate of digestion in the small intestine in comparison with readily digestible starch [13]. However, another possible mechanism is the induction of gut hormone release, mainly glucagon-like peptide 1 (GLP-1) and peptide YY (PYY), through the stimulation of intestinal L-cells by short chain fatty acids (SCFA) produced during fermentation. This pair of gut hormones is known to induce satiety and to regulate energy expenditure through central nervous system actions [14,15]. GLP-1 is able to stimulate insulin secretion and inhibit glucagon secretion [16]. Meanwhile, PYY can inhibit appetite, reduce energy intake, and also enhance insulin secretion [17]. 

Native banana starch (NBS) obtained from unripe bananas (*Musa* (AAA Group) ‘Dwarf Cavendish’ produced in Tabasco, Mexico, was used in this study. This product is rich in RS and has been demonstrated to induce a reduction in glycemic and insulinemic responses in healthy subjects and in patients with diabetes [18]. In obese women without diabetes, the administration of 30 g/day of NBS during four weeks reduced insulin resistance in a similar degree as 850 mg/day of metformin during the same period [19]. Moreover, supplementation with 24 g/day of NBS for four weeks caused lower BW and increased insulin sensitivity in a group of women with T2D [20]. Despite the encountered beneficial effects of NBS in reducing BW, we do not know whether this substance influences satiety level. Thus, the objective of this study was to evaluate the effects of acute NBS supplementation on subjective feelings of appetite, voluntary food intake, and gut hormonal response in a group of lean and overweight healthy subjects.

## 2. Materials and Methods 

### 2.1. Study Subjects 

The subjects were recruited through fliers placed around the División Académica de Ciencias de la Salud of the Universidad Juárez Autónoma de Tabasco. The experimental protocol was approved by the Ethical Committee of the Universidad Juárez Autónoma de Tabasco in compliance with the ethical principles and guidelines for the protection of human subjects under research. The purpose and risks of the study were explained to the participants before they provided written informed consent. 

Twenty-eight subjects were included in the study. All were healthy adult males and females, aged 18–25 years, and had a body mass index (BMI) between 18 and 29.9 kg/m^2^ and a waist circumference <85 cm (females) or <95 cm (males). Participants were selected based on a previous medical interview, anthropometric indexes, and basic laboratory examinations. Subjects not included in this study were those with a history of gastrointestinal disease or endocrine disorders, a history of cigarette smoking or alcoholism, vegetarians, a BMI ≥ 30, a diagnosis of diabetes or fasting glycemia >100 mg/dL, or chronic diseases such as renal or hepatic disorders as well as those that were pregnant, under psychiatric treatment, receiving medical or naturist treatment known to reduce BW or to affect appetite, or practicing intense physical activity (>60 min per day).

### 2.2. Study Design and Protocol

The study had a randomized single-blind crossover design. Two different beverages containing NBS or digestible corn starch (DCS) were tested on two different days separated by at least one week. The subjects were randomly assigned to the two different treatments. Prior to the test days, the participants followed a standardized fasting procedure: abstention from alcohol, caffeine, and hard physical activity for 24 h. Moreover, on the evening before the two test days, they were instructed to consume an identical evening meal no later than 8 PM.

On each of the 2 study days, subjects arrived at the Investigation Unit at 8:00 AM. Appetite sensation was assessed using visual analogue scales (VAS) at this time (time point 0). An intravenous cannula was inserted into an antecubital vein and a fasting blood sample was taken (0 min). Then the test products were served. Subjects received either 40 g NBS (70.5% RS type 2) or 40 g DCS (94.4% rapidly digestible starch), each dissolved in 200 mL of pure water. The two treatments were given in a pure form to avoid interference from other nutrients. After this, appetite sensation was assessed every 30 min for 180 min and serial blood samples were also taken postprandially at these same time points. 

During the experimentation period of each test day, subjects remained at the Investigation Unit and were allowed to read, use their computer, watch television, or talk as long as the conversation did not involve food. At the end of the intervention (180 min), subjects were placed in individual areas and provided with an ad libitum homogenous test meal to measure voluntary food intake. The buffet meal was pasta Bolognese that was served with 200 mL of pure water. The participants were instructed to drink all of the water and to eat until feeling comfortably satiated. The meal had a macronutrient composition of 56.12% of energy from carbohydrates, 28.18% from proteins, and 15.78% from fat, and an energy content of 154.58 kcal/100 g. Food was weighed before subjects sat down to the meal and reweighed after the subjects finishing eating to allow calculations of energy and macronutrient intake. The same type and amount of food was offered to each participant on both study days.

### 2.3. Test Products

Native banana starch (NBS) was obtained from unripe (green) bananas (*Musa* (AAA Group) ‘Dwarf Cavendish’ from a fruit-packing plant located at km 43.5 of the Villahermosa–Teapa highway in the Mexican state of Tabasco. The bananas, with a physiological age of 15 weeks, were isolated by means of a previously described procedure with some modifications [21]. Briefly, after washing, the bananas were peeled, cut into 5–6-cm pieces, immediately rinsed in a citric acid solution, and then macerated at low speed in an industrial blender for 4 min. The homogenate was sieved through screens (100, 200 US mesh) and washed with distilled water; it was then centrifuged at 10,000 rpm. The sediment was further purified by washed and centrifugation. The white starch sediment was dried in an air furnace at atmospheric pressure at 50 °C, passed through a 100-mesh sieve, and stored at room temperature in sealed glass jars. Proximate analysis included the following: 1.91% of moisture content; 2.05% protein; 0.57% fat, and 0.47% ash (14.003, 14.057, 14.059, and 14.006, AOAC international recommended methods). Resistant starch (RS) content was found to be 70.5% on a dry weight (DW) basis using a test kit (Megazyme© International, Wicklow, Ireland), accepted by the AOAC International (2002.02) and AACC International (32-40.01). The total starch content was 95% on DW basis. 

Digestible corn starch (DCS) was purchased from Unilever de México, S. de R.L. de C.V. as Maizena^®^ containing 94.4% rapid digestible starch.

An NBS 40 g sample contains 26.8 g of resistant starch and 9.32 g digestible starch, whereas DCS treatment contains 37.8 g of digestible starch. Considering that RS has only 2.5 kcal compared to 4 kcal for the DCS, the total energy content was 104.3 kcal for the NBS and 151.2 kcal for the DCS beverage. 

### 2.4. Appetite Assessment

To assess appetite sensation, visual analogue scales (VAS) were used. These were all 100 mm in length and anchored with words at each end, expressing the most positive and the most negative rating. Hunger, satiety, fullness, and prospective food consumption were assessed. Questions were asked as follows: (1) How strong is your desire to eat? (2) How hungry do you feel? (3) How full do you feel? (4) How much food do you think you could eat? Satiety is understood as between-meal satiety, which refers to the state of inhibition of eating, and fullness as the sensation of the degree of stomach feeling [22]. The use of VAS to assess subjective appetite sensation has been validated for its employment in postprandial single-meal studies [23].

### 2.5. Biochemical Measurements

Blood samples were collected in different tubes according to the required determination-to- perform. For common biochemical determinations, serum or EDTA plasma samples were obtained. For GLP-1, whole blood was collected into tubes containing K_3_-EDTA at a final concentration of 1.735 mg/mL. For PYY measurement, AEBSF [4-(2-aminoethyl) benzenesulfonyl fluoride hydrochloride] was used as a serine protease inhibitor. Serum and plasma were immediately frozen and stored at −70 °C until biochemical determination. Glucose, cholesterol, triglycerides, and insulin concentrations were performed using the Architect Clinical Chemistry Autoanalyzer System from Abbott Laboratories (Chicago, IL, USA). Glucose was determined by enzymatic assay with an imprecision of 5% total CV. Insulin was measured using chemiluminescent microparticle immunoassay (CMIA). Insulin assay imprecision was <7% of the total CV. Serum total PYY (1–36 and 3–36) and GLP-1 concentration were determined by an enzymatic-linked immunosorbent assay (Millipore Corporation Pharmaceuticals, St. Charles, MO, USA). All samples were batch analyzed by the same researcher within a single assay at the end of the study to eliminate interassay variability.

### 2.6. Statistical Analysis

In order to detect a difference of 10% in mean appetite ratings with a power >0.8 when using VAS [23], a group of 28 participants were included. To compare results of VAS scores between treatments, data were expressed as absolute (mm VAS) changes from the baseline (0 min). Data are expressed as means ± standard error of the mean (SEM) values, unless otherwise specified. Given the crossover design of the study, each participant represents his own control. The D’Agostino–Pearson normality test was performed to assess whether the data were consistent with Gaussian distribution. Time-course data were analyzed by a two-way repeated measures analysis of variance (ANOVA) to assess the effect of treatment, time, and interaction of treatment and time. Interpretation of findings were based on results from the ANOVA and not on the individual time point differences. A two-tailed paired Student *t* test was employed for comparisons between energy intakes from the same patients with different treatments. Differences were considered as statistically significant for *p* values < 0.05. Data were processed and analyzed using GraphPad Prism ver. 6.00 statistical software (GraphPad Software, Inc., San Diego, CA, USA). 

## 3. Results

### 3.1. Characteristics of Patients

Twenty-eight young participants (14 men and 14 women) completed the study. The majority of the subjects (16 subjects, 57.14%) exhibited as slightly overweight according to World Health Organization (WHO) criteria (BMI values >25 but <30). Waist circumference (WC) and waist-hip ratio (WHR) values corresponded to the normal range in both genders according to standards for Mexican population (women 0.71–0.85 m and men 0.78–0.94 m). All of the participants exhibited fasting normoglycemia according to the American Diabetes Association (ADA), with glycemia values <100 mg/dL. Cholesterol and triacylglycerols did not show abnormal levels according to the national and international reference range (Table 1). Both supplements were well tolerated by the subjects and no adverse gastrointestinal effects were reported on either of the test days. According to the crossover design utilized in this study, each subject received both treatments, NBS and DCS, in a random assignation process.

### 3.2. Subjective Appetite Measures 

**Figure 1:**
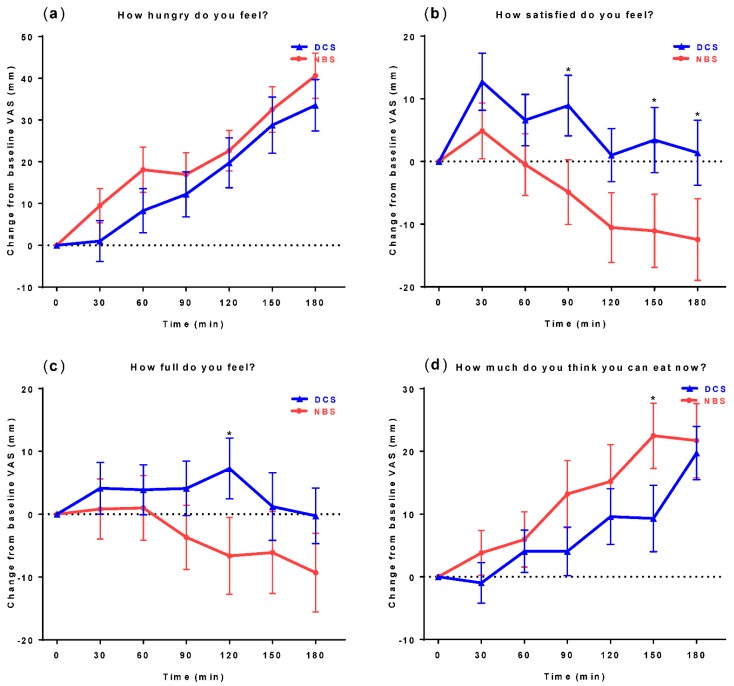
Effect of two beverages containing digestible corn starch (DCS) or native banana starch (NBS) on subjective appetite ratings of the visual analogue scale (VAS) questions (**a**) Hungry; (**b**) Satiety; (**c**) Fullness; and (**d**) Prospective consumption. Data are presented as change from baseline and expressed as mean ± standard error of the mean (SEM) for healthy subjects (*n* = 28). * Significant difference between groups (*p* < 0.05); repeated measures ANOVA with Tukey post hoc test.

Visual analogue scale measurements are illustrated in Figure 1. The absolute change scores for the desire to eat, hunger, fullness, and prospective food consumption increased significantly over time (*p* < 0.05), but no significant differences were observed between NBS and DCS supplementation after the two-way repeated measures ANOVA analysis. A trend to exhibit higher satiety, higher fullness, and lower prospective desire to eat was observed in the DCS group compared to the NBS group (Satiety: *t*90—*p* < 0.05, *t*150—*p* < 0.01, and *t*180—*p* < 0.05; fullness: *t*120—*p* < 0.05, and prospective desire to eat: *t*150—*p* < 0.05). 

### 3.3. Energy Intake

**Figure 2:**
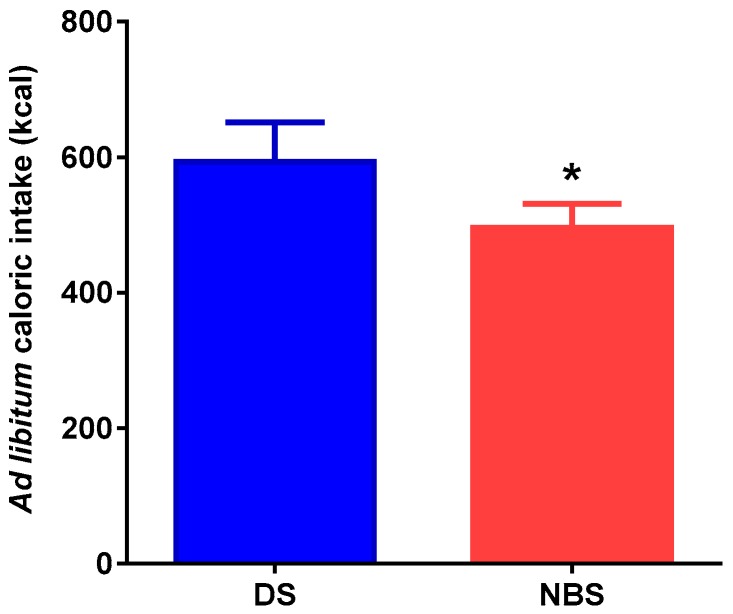
Total energy intake at the ad libitum homogenous test meal. The meal had a macronutrient composition of 56.12% of energy from carbohydrates, 28.18% from proteins, and 15.78% from fat, and an energy content of 154.58 kcal/100 g. Values represent mean ± standard error of the mean (SEM) for healthy subjects (*n* = 28). * Significant differences *p* < 0.05, paired Student *t* test.

Data on energy intake for NBS and DCS supplementation at the ad libitum test meal performed 180 min after intake of the test products are presented in Figure 2. Supplementation with NBS resulted in a significantly lower energy intake compared to the energy intake observed with the DCS supplement (3102 ± 1609.2 vs. 2591.6 ± 993.1; *p* = 0.0326).

### 3.4. Plasma Glucose and Insulin Levels

**Figure 3:**
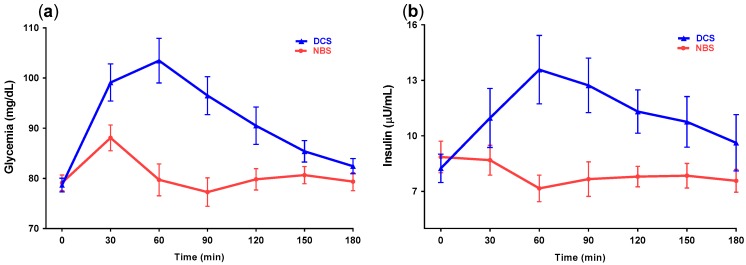
The effect of two beverages containing digestible corn starch (DCS) or native banana starch (NBS) on postprandial concentration of glucose (**a**) and insulin (**b**) in lean and overweight healthy subjects (*n* = 28). Data are expressed as mean ± standard error of the mean (SEM) and compared using repeated measures ANOVA with Tukey post hoc test.

A reduction in glycemic and insulin responses after NBS was observed (Figure 3). In two-way repeated measures ANOVA analysis for glycemia, there was a significant treatment (*p* = 0.0008) and time x treatment effect (*p* < 0.0001), where the NBS resulted in significantly lower glucose concentrations from 30 to 120 min after the meal compared to DCS. In ANOVA analysis for insulin, NBS resulted in a significant time x treatment factor (*p* = 0.0154) and lower insulin concentrations from 60 to 90 min.

### 3.5. Hormone Responses 

**Figure 4:**
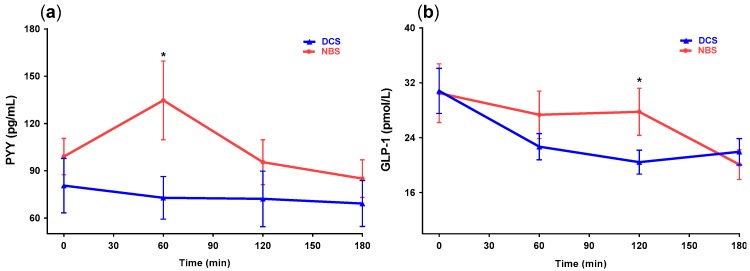
The effect of two beverages containing digestible corn starch (DCS) and native banana starch (NBS) on postprandial concentration of peptide YY (**a**) and GLP-1 (**b**) in lean and overweight healthy subjects (*n* = 28). Data are expressed as mean ± standard error of the mean (SEM) and compared using repeated measures analysis of variance (ANOVA) with Tukey post hoc test.

Figure 4 depicts gut hormone responses to supplementation of the different starches. In two-way repeated measures ANOVA analysis, no difference was observed in the postprandial PYY response according to treatment, but a significant time x treatment effect was observed (*p* = 0.0266); at 60 min, the NBS exhibited higher PYY concentrations compared to DCS (*p* < 0.0001). In GLP-1 response, no difference was observed between treatments; however, a time x treatment effect was observed (*p* = 0.0096), where GLP-1 level was significantly higher at 120 min compared to DCS. 

## 4. Discussion

In this study, we demonstrated that the acute ingestion of a beverage containing 26.8 g RS from unripe bananas reduced postprandial glucose, insulin response, and subsequent voluntary energy intake. However, there was no associated effect on subjective appetite ratings. To our knowledge, this is the first study to analyze the acute effects of banana starch on voluntary food intake and subjective appetite ratings as the majority of related studies have used RS from other sources.

In the present study, a reduction in glycemic and insulinemic responses was observed following RS treatment in comparison with DCS treatment. This can be partially explained by the differences in the digestibility of the treatments. Banana starch, classified as RS2, is protected from enzymatic digestion by its compact conformation, which consists of amylopectin crystals of uncooked native-starch granules, and is usually in B- or C-type crystalline polymorph. In contrast, DCS contains a less ordered structure and is more easily hydrolyzed by digestive enzymes [11]. As a result of its indigestibility, RS only provides around 2.5 kcal/g; this amount is slightly more than half the caloric value of digestible starch, which produces 4.0 kcal/g [24]. The observed reduction in glycemic response confirms previous studies and highlights the benefit of including RS in food preparation to help control glycemia. Post-meal hyperglycemia is considered a risk factor for cardiovascular disease, even in subjects without diabetes, and its regulation is more important than controlling fasting glycemia in subjects with diabetes in order to achieve optimal HbA1c targets [25,26,27].

Notably, our results demonstrated that NBS supplementation reduced voluntary food intake upon experimental manipulation in comparison to DCS supplementation. The ad libitum test meal used in this study is the best method to evaluate satiation, which is the process that leads to cessation of eating, and determines meal size [28]. Considering that one important factor contributing to obesity is overconsumption of energy, the use of meals supplemented with NBS may help to reduce meal size and to control BW. Unexpectedly, there was no correlation in this study between reduced voluntary food intake and subjective changes in hunger, appetite, satiety, and fullness. In this respect, the results on the effects of RS from different sources on subjective markers of appetite have been inconsistent. While many researchers have found that RS has no effect on appetite markers [29,30,31,32], others have reported a reduction in satiety [33,34,35].

In addition, our results showed that serum glucose and insulin were not correlated with subjective appetite ratings, although a trend of higher satiety was observed during the late-postprandial period after DCS consumption. According to the glucostatic theory, high-glycemic responses are associated with greater satiety in the short term, whereas falling blood glucose concentrations that occur several hours after a high-glycemic meal are associated with less satiety [36,37]. In fact, some researchers have found high insulin levels to be more important for increasing satiety than high glycemia. However, increased glycemia clearly precedes insulin secretion. Flint et al. reported insulin to be positively associated with increased satiety and decreased hunger in a group of normal weight subjects [38]. Insulin can affect appetite by acting on hypothalamic neurons, promoting anorexia by decreasing NPY and stimulating POMC expression and through regulating ghrelin suppression [39,40].

In summary, the appetite scores of the present study do not support the glucostatic theory, but the reduction in caloric ingestion after NBS consumption is in accordance with this hypothesis since the treatment with the lower glycemic and insulinemic responses also induced a reduction in voluntary food intake. At present, the relationship between high glycemic response and food intake remains controversial; some researchers have shown a direct correlation between meals with high glycemic response and voluntary food intake [36,37,41,42,43], while others have found no correlation [44,45,46].

The aim of this study was to investigate the effects of NBS on subjective appetite ratings, food intake, and gut hormones. For this purpose, we used similar quantities of different starches (NBS and DCS) mixed in pure water to avoid interference from other nutrients. Thus, the treatments were only matched for starch weight and not for available carbohydrates or energy content. In a comparable acute study that matched starch weight, 50 g of raw potato starch (27.1 g of RS) and 50 g of pregelatinized potato starch (100% digestible) were administered to healthy lean subjects. These results were similar to those of the present study: after RS treatment, a reduction in postprandial glycemia and insulin occurred [47]. In that study, however, diminished subjective scores for satiety and fullness were observed, and voluntary food intake or gut hormones were not determined. In another study in which treatments were matched by available carbohydrates, the effect of 48 g of RS from high amylose maize on healthy males was studied. Although, RS did not modify postprandial glycemia and no associated effect was observed on subjective appetite scores, a lower postprandial insulin response and a reduction in energy intake were observed [29]. 

The modulation of gut hormones is one potential mechanism that could explain the benefits of RS for glycemic metabolism and satiety in addition to low digestibility. The increase of serum GLP-1 and PYY after RS consumption has been related to the production of short-chain fatty acids (SCFA) by fermentation and the interaction of these substances with free fatty acid receptors 2 and 3 (FFAR2 and FFAR3) in intestinal L-cells [48]. However, in the present study, no differences between treatments were observed according to the two-way repeated measures ANOVA, and only a moderate increment was observed in GLP-1 at 120 min, with values subsequently falling at 180 min. Findings from other acute studies in humans have been inconsistent. Bodinham et al. using 48 g of RS2, reported a reduction of GLP-1 compared to the control group [49]. Klosterbauer et al. found that subjects consuming 25 g of RS3 had a lower postprandial GLP-1 level [31]. The majority of studies reporting increased blood gut hormones after the ingestion of indigestible carbohydrates have been conducted over long-term treatment periods that allow the fermentation process to be carried out [50,51,52]. There is a need for studies of longer duration in order to understand the beneficial effects of RS and the participation of gut hormones. Other potential pathways by which RS can improve metabolic health include modulation of gut microbiota, circulating inflammatory mediators, innate immune cells, and the bile acid cycle [12]. However, these mechanisms also could be operating in either the short or the long term. 

This study had many strengths. A sufficiently large sample size (*n* = 28) was included to enable a reliable analysis with appropriate statistical power. The study recruited an equal number of male and female subjects and a sufficient number of normal weight and overweight participants. The inclusion of a crossover design reduces the influence of confounding covariates because each patient serves as his or her own control. However, several limitations of our study need to be mentioned. First, our study could not be double blinded because different flavored beverages were used. The study was performed in lean and overweight subjects; thus, results cannot be extended to patients with other metabolic disturbances. We did not measure hydrogen production to estimate the fermentation process; finally, we did not measure serum ghrelin, which is considered to be the first hormone that acts on appetite initiation.

In conclusion, in healthy, lean, and overweight subjects, the supplementation of 28 g of RS from bananas reduced voluntary energy intake and glycemic and insulinemic responses; however, no association was observed with subjective appetite ratings or gut hormone responses. These effects could have important implications for body weight control and the improvement of postprandial metabolism in lean and overweight subjects. More studies are needed to clarify the complex mechanisms underlying the relationships between RS supplementation and the appetite-regulating process.

## Figures and Tables

**Table 1:** Baseline characteristics of the study subjects.

Characteristic	Female	Male	Total
*n* of subjects	14	14	28
Gender (M/F) %			50/50
Age (years)	23 (23, 23)	20.5 (19, 21.25)	21.75 ± 1.92
Height (cm)	158 ± 7.34	170.3 ± 7.30	164.1 ± 9.53
Body weight (kg)	59.16 ± 8.79	72.61 ± 11.47	65.89 ± 12.14
BMI (kg/m^2^)	23.67 ± 2.57	25.04 ± 3.28	24.36 ± 2.97
Waist circumference (cm)	76.57 ± 6.98	85.96 ± 8.20	81.27 ± 8.87
Waist-to-hip ratio	0.78 ± 0.05	0.85 ± 0.04	0.82 ± 0.06
Glycemia (mg/dL)	83.57 ± 5.87	82.64 ± 9.90	83.11 ± 8.00
Total cholesterol (mg/dL)	168.1 ± 33.76	172.6 ± 27.78	170.3 ± 30.43
Triglycerides (mg/dL)	89 (72.5, 161.5)	92.5 (65.75, 126.5)	92.50 (69.50, 153.8)

All values are expressed as mean ± standard deviation (SD) or median (25, and 75 percentiles). BMI = body mass index.
